# Snowbirds and infection--new phenomena in pneumonia and influenza hospitalizations from winter migration of older adults: A spatiotemporal analysis

**DOI:** 10.1186/1471-2458-11-444

**Published:** 2011-06-07

**Authors:** Kenneth KH Chui, Steven A Cohen, Elena N Naumova

**Affiliations:** 1 Department of Public Health and Community Medicine, Tufts University School of Medicine, Boston, Massachusetts, USA; 2 Department of Civil and Environmental Engineering, Tufts University School of Engineering, Medford, Massachusetts, USA

## Abstract

**Background:**

Despite advances in surveillance and prevention, pneumonia and influenza (P&I) remain among the leading causes of mortality in the United States. Elderly adults experience the most severe morbidity from influenza-associated diseases, and have the highest rates of seasonal migration within the U.S. compared to other subpopulations. The objective of this study is to assess spatiotemporal patterns in influenza-associated hospitalizations in the elderly, by time, geography, and intensity of P&I. Given the high seasonal migration of individuals to Florida, this state was examined more closely using harmonic regression to assess spatial and temporal patterns of P&I hospitalizations by state of residence.

**Methods:**

Data containing all Medicare-eligible hospitalizations in the United States for 1991-2006 with P&I (ICD-9-CM codes 480-487) were abstracted for the 65+ population. Hospitalizations were classified by state of residence, provider state, and date of admissions, specifically comparing those admitted between October and March to those admitted between April and September. We then compared the hospitalization profile data of Florida residents with that of out-of-state residents by state of primary residence and time of year (in-season or out-of-season).

**Results:**

We observed distinct seasonal patterns of nonresident P&I hospitalizations, especially comparing typical winter destination states, such as California, Arizona, Texas, and Florida, to other states. Although most other states generally experienced a higher proportion of non-resident P&I during the summer months (April-September), these states had higher nonresident P&I during the traditional peak influenza season (October-March).

**Conclusions:**

This study is among the first to quantify spatiotemporal P&I hospitalization patterns in the elderly, focusing on the change of patterns that are possibly due to seasonal population migration. Understanding migration and influenza-associated disease patterns in this vulnerable population is critical to prepare for and potentially prevent influenza outbreaks in this vulnerable population.

## Background

Over the past several decades, influenza prevention has benefitted from advances such as viral sequencing and the formulation and distribution of vaccine [1]. Despite national and global preventive efforts, influenza still poses a challenge to the provision of medical care. In fact, influenza and pneumonia, a common manifestation, consistently remain in the top ten causes of death nationwide [2].

Although the dynamics of influenza are well documented [3], mechanisms of such dynamics are poorly understood [4,5]. There is substantial inter-seasonal variation in the geographical distribution of disease, timing, severity, and the population groups most affected. Influenza occurs throughout the United States non-uniformly [6]; some influenza strains demonstrate distinct inter-hemispheric synchrony and latitudinal gradients in epidemic peaks [7]. The timing of the seasonal peak in influenza changes annually, ranging from 24^th ^to 33^rd ^Julian calendar day [3]. Together, space and timing of the seasonal peak work in synchrony to dictate the epidemiological effects of influenza in the population [8]. The severity of influenza also varies by individual influenza season [9].

Accurate monitoring and estimation of influenza activity require understanding not only influenza dynamics, but also population characteristics and their changes. Important dynamic factors such as the transmissibility of influenza strains, the accuracy of contact rate assessment, and the movement of populations within and between geographical units, if not clearly understood and properly modelled, may compromise the precision and accuracy of the estimation. The SARS epidemic of 2003 [10] illustrates these complexities by demonstrating the potential of global travel patterns to impact the spread of a virulent infectious agent, the coronavirus, necessitating a systematic investigation on how travel and migration patterns affect disease epidemics.

Although some historical influenza epidemics and pandemics disproportionately affect the younger population, such as the 1918 Spanish influenza [11] and the recent H1N1 pandemic of 2009 [12], for most seasonal influenza, the elderly (defined as the population aged 65 and above) are the most severely affected. From 1990 to 1998, over 90% of influenza-associated mortality occurred in the elderly, much of which from influenza-associated pneumonia co-infection. Influenza-associated morbidity is highest in the elderly. Patients with a primary diagnosis of pneumonia accounted for nearly 700,000 average annual hospitalizations from 1998 to 2002. There were over 1.2 million hospitalizations in the elderly with any-listed diagnosis of pneumonia during that period [13].

The elderly, who experience the most severe morbidity and highest mortality from influenza, are also one of the most mobile population groups due to their frequent seasonal migrations. Popular destinations in the U.S. include Florida, Arizona, and Texas. These migrations can be sizable: in 2005, the proportion of non-permanent elderly residents increases from 0.5% in September (summer time in the US) to 12.1% in January [14]. Unlike younger populations who may travel temporarily for work and leisure, the elderly tend to stay for longer periods of time and use more health services [15]. These migrations may affect influenza transmission and, as a consequence, change health care service utilization patterns both at their home state and in their temporary residence or lodging [14]. Evidence suggests that hospitalization patterns for in-state residents differ from temporary residents [16]. This has important implications, not only by potentially affecting the dynamics and transmission patterns of influenza itself, but also by impacting health care service providers. Peak seasonal migration to Florida and other southern states coincides with the typical peak in influenza, which generally occurs in the winter months [3,14]. There are few studies that have assessed differences in patterns of influenza-related hospitalization in the Medicare population simultaneously by time of year, provider state, and state of residence.

The purpose of this analysis is to quantify these spatiotemporal patterns in influenza-associated hospitalizations in the elderly using Medicare-associated hospitalization claims in the United States for 16 years, including 15 complete influenza seasons (July 1991-June 2006). We compare spatiotemporal patterns of influenza activity across all states of the U.S., emphasizing the simultaneous comparison of seasonal migration patterns by season. We then focus the analysis on Florida, a state known for extensive seasonal migration of elderly adults, to examine how seasonal migration to Florida affects the timing, geography, and intensity of pneumonia and influenza across seasons.

## Methods

### Data source and preparation

We abstracted 21.5 million out of 229.7 million hospitalization records obtained from Centers for Medicare and Medicaid Services (CMS) based on a set of previously established entry criteria [3]: admission happened from 1991 through 2006, patient's age was 65 or above when admitted, and the patient's diagnoses includes pneumonia and influenza (P&I; International Classification of Diseases, Ninth Revision, Clinical Modification codes 480-487). Variables used in this analysis are patient's age, date of admission, state of residence, and state in which the patient was hospitalized, referred to as "provider state" hereafter.

For the national state-level analysis, we compiled cumulative frequencies of the P&I hospitalization in two time periods--October 1^st ^through March 31^st ^and April 1^st ^through September 30^th^--approximating the in- and off-seasons. For the analysis on Florida, we created a 5884-day-long time series by compiling daily counts of the P&I hospitalization for Floridians and non-Floridians.

### Data analysis

The first part of the analysis assessed the spatiotemporal patterns of influenza-associated hospitalizations for all 50 states plus the District of Columbia by comparing the ratios of hospitalized residents to hospitalized non-residents between the two time periods with χ^2 ^tests. To better explore the relative changes in rates and counts of P&I hospitalizations in specific state of residence and provider state, we visualized these multivariate relationship using bubble matrix plots [17]. As an extension of the aforementioned χ^2 ^analysis, a second bubble matrix was made to show the natural log transformed ratio of the number of non-Floridians to the number of Floridians for each combination of the provider states and states of residence.

The second part of the analysis focused on understanding the residential makeup and temporal fluctuation of the elderly hospitalized in Florida. Demographic characteristics of the two groups (Floridian vs. Non-Floridian) were compared with t-test and χ^2 ^test. The original states of residence of the non-Floridian were tabulated. The counts were then segregated by the two periods of time for calculating the seasonal ratios (frequency of hospitalizations in April-September divided by frequency of hospitalizations in October-March). The resultant ratios were visualized using mapping.

To understand the temporal differences between the two residential groups, time-series plots were created illustrating the change in the hospitalization counts between the two groups. We then used harmonic regression to estimate the times to peak for each group, controlled for long-term trends. The general equation of the harmonic regression is as follows:

where ln[E(Counts)] are hospitalization counts modelled with Poisson distribution, π is the constant, ω is the frequency, and t is the day in a time series ranging from 1^st ^to 5884^th ^day. The terms β_3_(t) and β_4_(t^2^) control for long-term linear and quadratic trends. The coefficients β_1 _and β_2 _are needed to derive peak timing in days. Detailed methods can be found elsewhere [18].

SAS version 9.0 (Cary, NC) and S-PLUS version 8 (Palo Alto, CA) were used for the data abstraction and analysis, respectively. ArcGIS version 9.3 (Redlands, CA) was used for mapping. The Tufts Medical Center Institutional Review Board approved the study protocol for this analysis of the CMS data.

## Results

### National Pneumonia and Influenza Hospitalizations by State

For the majority of states, a significantly higher proportion of non-resident P&I hospitalizations occurred from April-September than from October-March (Table 1). Significantly higher proportions of non-resident P&I hospitalizations occurred from October-March than in April-September in a minority of states--Arizona, California, Florida, Hawaii, Nevada, South Carolina, and Texas.

**Table 1:** Number (percent) of all seasonal pneumonia and influenza hospitalizations by time of year, state of residence, and provider state, 1991-2006

State	October-March		April-September		High	P-value*
	
	Residents	Nonresidents	Residents	Nonresidents	season	
**Alabama**	248632 (96.3)	9472 (3.7)	172927 (96.4)	6429 (3.6)	Oct-Mar	0.138

**Alaska**	9188 (96.2)	363 (3.8)	7914 (88.5)	1033 (11.6)	Apr-Sep	< 0.001

**Arizona**	142096 (84.7)	25635 (15.3)	94299 (91.9)	8371 (8.2)	Oct-Mar	< 0.001

**Arkansas**	165858 (92.9)	12749 (7.1)	113955 (92.8)	8621 (7.2)	Oct-Mar	0.626

**California**	845466 (97.0)	25685 (3.0)	604817 (97.4)	15907 (2.6)	Oct-Mar	< 0.001

**Colorado**	108513 (94.4)	6419 (5.6)	76255 (93.2)	5568 (6.8)	Apr-Sep	< 0.001

**Connecticut**	156200 (95.1)	8069 (4.9)	116421 (94.6)	6616 (5.4)	Apr-Sep	< 0.001

**Delaware**	28834 (92.3)	2403 (7.7)	21747 (91.8)	1955 (8.2)	Apr-Sep	0.017

**D.C**.	18095 (69.0)	8133 (31.0)	14086 (68.6)	6441 (31.4)	Apr-Sep	0.392

**Florida**	617641 (89.4)	73042 (10.6)	465872 (94.1)	28981 (5.9)	Oct-Mar	< 0.001

**Georgia**	332552 (94.6)	18841 (5.4)	229626 (94.6)	13076 (5.4)	Oct-Mar	0.664

**Hawaii**	26353 (93.3)	1893 (6.7)	21669 (95.6)	1007 (4.4)	Oct-Mar	< 0.001

**Idaho**	43865 (93.9)	2848 (6.1)	30702 (92.4)	2542 (7.6)	Apr-Sep	< 0.001

**Illinois**	589003 (97.3)	16151 (2.7)	438403 (97.0)	13505 (3.0)	Apr-Sep	< 0.001

**Indiana**	300301 (93.7)	20074 (6.3)	222693 (93.2)	16143 (6.8)	Apr-Sep	< 0.001

**Iowa**	179608 (93.8)	11839 (6.2)	126674 (93.5)	8885 (6.5)	Apr-Sep	< 0.001

**Kansas**	165757 (94.7)	9206 (5.3)	110418 (94.4)	6537 (5.6)	Apr-Sep	< 0.001

**Kentucky**	281541 (94.7)	15631 (5.3)	203019 (94.5)	11801 (5.5)	Apr-Sep	< 0.001

**Louisiana**	251425 (96.4)	9437 (3.6)	174827 (96.4)	6519 (3.6)	Oct-Mar	0.689

**Maine**	59499 (97.3)	1661 (2.7)	43596 (94.9)	2446 (5.3)	Apr-Sep	< 0.001

**Maryland**	190971 (92.1)	16361 (7.9)	141807 (91.8)	12646 (8.2)	Apr-Sep	0.001

**Massachusetts**	311878 (95.2)	15664 (4.8)	229926 (94.3)	13856 (5.7)	Apr-Sep	< 0.001

**Michigan**	405840 (98.1)	7921 (1.9)	314254 (97.3)	8691 (2.7)	Apr-Sep	< 0.001

**Minnesota**	192294 (94.3)	11529 (5.7)	141106 (92.9)	10727 (7.1)	Apr-Sep	< 0.001

**Mississippi**	169578 (95.3)	8414 (4.7)	114003 (95.1)	5874 (4.9)	Apr-Sep	0.030

**Missouri**	333352 (93.1)	24737 (6.9)	235351 (92.6)	18720 (7.4)	Apr-Sep	< 0.001

**Montana**	44327 (96.5)	1618 (3.5)	30941 (94.6)	1754 (5.4)	Apr-Sep	< 0.001

**Nebraska**	93004 (94.3)	5647 (5.7)	65457 (93.5)	4582 (6.5)	Apr-Sep	< 0.001

**Nevada**	52948 (86.4)	8308 (13.6)	38742 (87.0)	5801 (13.0)	Oct-Mar	0.011

**New Hampshire**	40253 (88.1)	5431 (11.9)	28592 (86.4)	4506 (13.6)	Apr-Sep	< 0.001

**New Jersey**	331599 (96.0)	13835 (4.0)	251794 (95.5)	11942 (4.5)	Apr-Sep	< 0.001

**New Mexico**	62858 (93.5)	4348 (6.5)	40350 (93.3)	2897 (6.7)	Apr-Sep	0.133

**New York**	702238 (97.4)	18964 (2.6)	542368 (96.8)	18176 (3.2)	Apr-Sep	< 0.001

**North Carolina**	361924 (95.9)	15539 (4.1)	256028 (95.4)	12213 (4.6)	Apr-Sep	< 0.001

**North Dakota**	41906 (86.3)	6661 (13.7)	31203 (85.5)	5261 (14.5)	Apr-Sep	0.002

**Ohio**	578674 (96.5)	21010 (3.5)	430959 (96.0)	17813 (4.0)	Apr-Sep	< 0.001

**Oklahoma**	219441 (96.7)	7469 (3.3)	150865 (96.6)	5341 (3.4)	Apr-Sep	0.031

**Oregon**	89788 (93.5)	6237 (6.5)	65729 (92.9)	5053 (7.1)	Apr-Sep	< 0.001

**Pennsylvania**	548327 (95.9)	23642 (4.1)	405591 (95.4)	19536 (4.6)	Apr-Sep	< 0.001

**Rhode Island**	42368 (92.5)	3438 (7.5)	30789 (91.6)	2832 (8.4)	Apr-Sep	< 0.001

**South Carolina**	162490 (96.0)	6712 (4.0)	113858 (96.2)	4517 (3.8)	Oct-Mar	0.040

**South Dakota**	57904 (91.4)	5435 (8.6)	40526 (90.5)	4278 (9.5)	Apr-Sep	< 0.001

**Tennessee**	345673 (92.3)	28957 (7.7)	250242 (91.9)	21961 (8.1)	Apr-Sep	< 0.001

**Texas**	770764 (95.9)	32772 (4.1)	523340 (96.4)	19517 (3.6)	Oct-Mar	< 0.001

**Utah**	55929 (93.9)	3667 (6.1)	39408 (92.9)	3024 (7.1)	Apr-Sep	< 0.001

**Vermont**	23032 (88.4)	3016 (11.6)	17270 (86.3)	2735 (13.7)	Apr-Sep	< 0.001

**Virginia**	252964 (94.1)	15905 (5.9)	183771 (93.8)	12102 (6.2)	Apr-Sep	< 0.001

**Washington**	162143 (95.4)	7884 (4.6)	121115 (94.8)	6606 (5.2)	Apr-Sep	< 0.001

**West Virginia**	138485 (89.7)	15479 (10.3)	100701 (89.3)	10287 (10.7)	Apr-Sep	< 0.001

**Wisconsin**	211858 (96.0)	8750 (4.0)	156389 (95.3)	7800 (4.7)	Apr-Sep	< 0.001

**Wyoming**	20341 (94.8)	1123 (5.2)	14451 (91.8)	1294 (8.22)	Apr-Sep	< 0.001


*χ*^2 ^test

**Figure 1:**
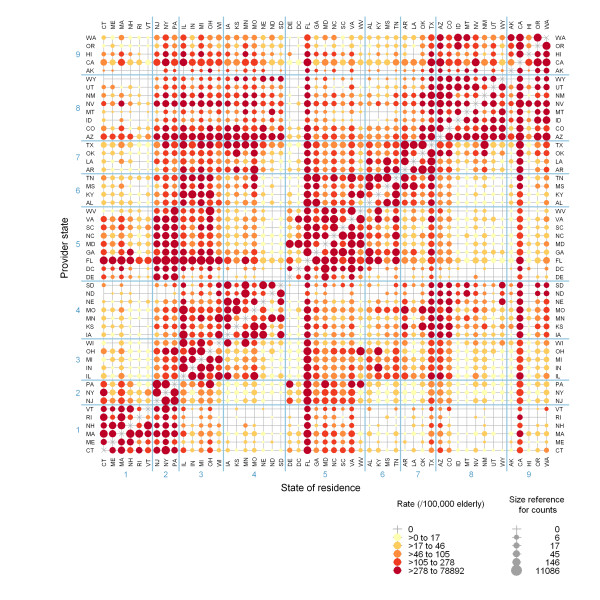
**Relative numbers and rates of pneumonia and influenza hospitalizations classified by state of residence and provider state, October-March**. The different hues represent rates and the sizes represent counts. Hospitalizations with same residential and provider codes (i.e. the diagonal line) were not visualized due to their big sizes. States are ordered by the nine Census divisions--1: New England; 2: Middle Atlantic; 3: East north Central; 4: West North Central; 5: South Atlantic; 6: East South Central; 7: West South Central; 8: Mountain; 9: Pacific.

Examining the non-resident P&I hospitalizations, we observe uneven patterns in the distribution of state of residence. Figure 1 depicts a bubble plot of the cross-classification of provider state and state of residence for all P&I cases from 1991 through 2006 from October through March. The size of the bubble represents frequencies of hospitalization and the hue represents rates. Information in the diagonal cells was omitted because those cells would be of much higher counts and rates, which is natural because most people are hospitalized in their own state, dwarfing the small but important distinctions among the discordant cells.

Residents of certain states have a greater propensity to be treated out-of-state for P&I than many other states. Non-residents of states such as California, Arizona, Florida, and Texas comprise a notable proportion of the total P&I hospitalizations treated in hospitals in these states. States are displayed alphabetically within Census divisions. In general, adjacent groups of states tend to have the largest discordances between provider state and state of residence for P&I cases. This is especially evident in several New England states, New York, New Jersey, and Pennsylvania, as well as many residents of Alabama being treated in neighbouring Georgia and Florida, and vice versa.

**Figure 2:**
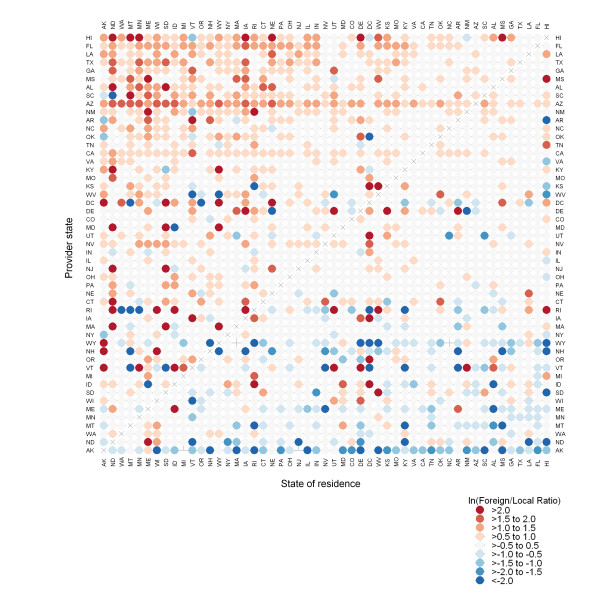
**Comparison of pneumonia and influenza hospitalization rate by time of year, state of residence, and provider state**. Orange dots represent areas where the rate of hospitalizations is greater between October and March than between April and September; blue dots represent states where the rate of hospitalization is less between October and March than between April and September. States are ordered by centroid latitude, from north to south.

There were notable similarities and differences between the typical high season for influenza--October through March--compared to the period of time between April and September, which generally has lower rates of disease. Figure 2 depicts the ratio of P&I hospitalizations comparing counts from October to March to counts from April to September, by provider state and state of residence. Orange dots represent states in which the rates are higher for October-March than for April-September. Blue dots represent states in which P&I rates are higher for April-September than for October-March. Higher colour saturations represent rate ratios of higher magnitude. Certain states, such as Hawaii, Arizona, Texas, and Florida show higher rates of non-resident P&I hospitalizations in October-March than for the time period of April-September for most states. The non-resident P&I cases in Arizona appear to come from a fairly even distribution of many other states. In contrast, out-of-state residents being hospitalized in Florida for P&I were derived primarily from northeastern, and Midwestern states. Many northern states had the opposite pattern entirely: the northernmost states--including Maine, Vermont, North Dakota, and Montana and others--actually experienced decreases in out-of-state resident P&I hospitalizations during the influenza season compared to the off-season.

### Example state: Florida

Examining the state of Florida more closely, there were 1,185,543 P&I hospitalization in Florida between 1991 and 2006. Of these 102,030 (9.4%) cases were associated with patients living out of Florida. Non-Floridian patients tended to be younger (79.5 vs. 80.3 years, p < 0.001, t-test), more likely male (52.9% male vs. 47.0%, p < 0.001, χ^2 ^test), and predominately white (94.4% vs. 87.7%, p < 0.001, χ^2 ^test). Detailed frequencies can be found in Table 2.

**Table 2:** Demographic characteristics of all pneumonia and influenza hospitalizations in Florida, 1991-2006

Variables	Florida residents	Non-Florida residents	Differences
Age, mean ± SD	80.25 ± 8.28	79.45 ± 8.00	p < 0.001^a^
Gender, count (%)			
Male	459761 (47.0%)	50941 (52.9%)	p < 0.001^b^
Female	518041 (53.0%)	45339 (47.1%)	
Race, count (%)			
White	857123 (87.7%)	90897 (94.4%)	
Black	69474 (7.1%)	2416 (2.5%)	
Asian	2336 (0.2%)	193 (0.2%)	p < 0.001^b^
Hispanic	30381 (3.1%)	887 (0.9%)	
North American Native	385 (0.04%)	53 (0.1%)	
Others	10168 (1.0%)	638 (0.7%)	

^a ^Independent-samples t-test

^b ^*χ*^2 ^test

**Figure 3:**
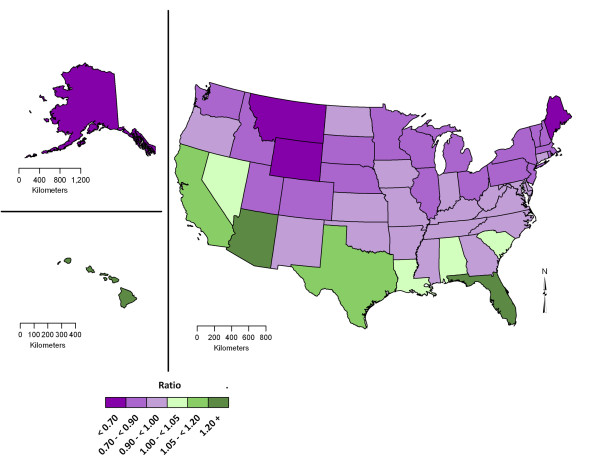
**State-specific ratios of hospitalizations associated with pneumonia and influenza happened in Florida in the period April-September to that in October-March, 1991-2006**.

A closer examination of the specific states from which the non-resident P&I cases hospitalized in Florida reveals that a large proportion of those individuals are residents of Northeast and Midwestern states (Table 3). The top six states provided the majority (53.7%) of all out-of-state cases: New York, Michigan, Ohio, Pennsylvania, New Jersey, and Georgia. The two states that share a border with Florida, Georgia and Alabama, provide 8.6% of all non-resident P&I cases. Furthermore, the mapping of the seasonal ratios (frequency in April-September/frequency in October-March) of non-Floridians hospitalized in Florida reveals a distinct spatial pattern (Figure 3). States shaded in purple provide more non-resident P&I hospitalizations to Florida during between October and March than between April and September. The darker the hue, the greater the difference is between October-March and April-September. In states shaded in green--namely California, Nevada, Texas, Louisiana, Alabama, South Carolina, Hawaii, and Florida itself, there were more non-resident P&I cases occurring in Florida during April-September than between October and March.

**Table 3:** Total number and state of residence of elderly hospitalized in Florida due to pneumonia and influenza, 1991-2006

State of residence	N	State of residence	N
Florida	1083513	Rhode Island	716
New York	15984	Iowa	620
Michigan	9703	Vermont	593
Ohio	6959	Louisiana	445
Pennsylvania	5944	Colorado	413
New Jersey	5705	Delaware	406
Georgia	5416	Arizona	403
Illinois	5066	Mississippi	400
Massachusetts	4915	Arkansas	369
Alabama	4157	Washington	357
Indiana	3706	Kansas	217
Connecticut	2658	Nevada	211
North Carolina	2214	Oklahoma	194
Maryland	2044	New Mexico	185
Virginia	1965	Oregon	171
Wisconsin	1896	District of Columbia	153
California	1642	Nebraska	138
Maine	1506	South Dakota	80
New Hampshire	1448	North Dakota	75
Tennessee	1445	Hawaii	74
Kentucky	1429	Idaho	73
Texas	1254	Alaska	69
Minnesota	1098	Utah	68
West Virginia	1011	Montana	64
South Carolina	978	Wyoming	61
Missouri	941	Other locations	4391

**Figure 4:**
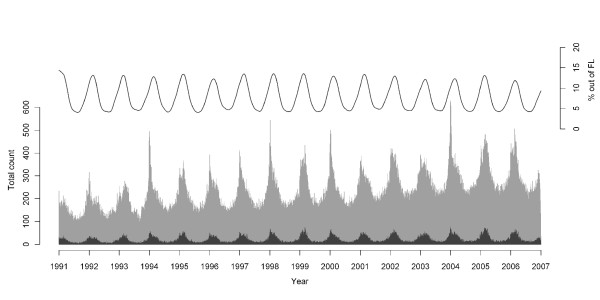
**Daily time-series plot of total counts of pneumonia and influenza hospitalizations in Florida by residential status, 1991-2006**. The grey bars represent Florida residents and black bars represent out-of-state residents. The line on the top represents the percentage of all pneumonia and influenza hospitalizations from out-of-state residents in Florida.

Examining all P&I cases occurring in Florida comparing residents to nonresidents, we observed distinct seasonality in the numbers of P&I hospitalizations attributable to out-of-state residents but who sought care in Florida (Figure 4). This graph illustrates the seasonal peaks in weekly counts of both the resident and non-resident P&I hospitalizations in Florida. There is also distinct seasonality in the percent of all P&I hospitalizations attributable to non-Florida residents. This percentage oscillated between approximately 4% during the seasonal nadirs in the summer months to 13% during the typical wintertime increases. According to the results of the harmonic regression, hospitalizations of the out-of-state patients peaked at about the third week of January, which is about one week later than that of the same-state patients (28^th^±0.005 Julian calendar day vs. 23^rd^±0.005, p < 0.001).

## Discussion

We found distinct, state-specific hospitalization patterns that differ across provider states and over time. In several states, such as California, Arizona, Texas, and Florida, among others, the proportion of non-residents being hospitalized for P&I was higher in the winter months than in the summer months, although for most states, the opposite was true. In Florida, the proportion of all P&I hospitalizations attributable to out-of-state residents was over three times as high between October and March compared to the usual nadir of influenza activity, April through September. A large portion of out-of-state resident P&I hospitalizations in Florida are derived from northeastern and Midwestern states, such as New York, Michigan, Pennsylvania, and Ohio. The patterns observed in Florida are similar to those observed in other destination states for seasonally migrating elderly, including Texas, California, and Arizona, except that the composition of states of primary residence are slightly different than that of Florida.

**Figure 5:**
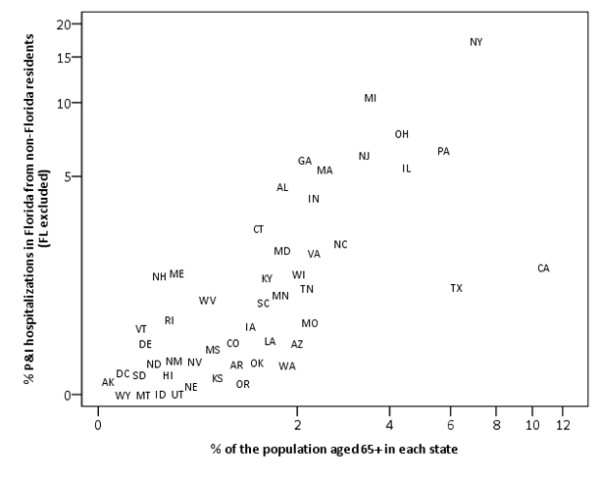
**Percent of all non-resident pneumonia and influenza hospitalizations occurring in Florida by state of residence and percent of all elderly (aged 65 or above) in each state, 1991-2006**.

Furthermore, the top contributors of hospitalized non-residents are not necessarily with the largest proportions of the national elderly population (Figure 5). Clear discrepancies exist between the proportion of all U.S. elderly living in the state and each state's contribution to non-resident P&I hospitalizations in Florida. Michigan, for example, has the eighth largest population of elderly in the nation, yet the state contributed the second-highest number of P&I cases to the non-resident P&I hospitalizations in Florida. Texas contains the fourth-highest number of elderly residents in the U.S. (2,072,532), yet contributed less than 1% of the total number of out-of-state P&I hospitalizations in Florida during the period of study. Similarly, California, the state with the largest elderly population counts in the nation, has 10.2% of the entire elderly population. However, the state provided only 1.6% of all out-of-state P&I hospitalizations in Florida. These findings suggest substantive difference in seasonal migration patterns of elderly individuals on a state-by-state basis.

National data on seasonal migration of the elderly within the United States are not readily available. Estimates of seasonal migration are available only through proxies or surveys [14]. Our findings contribute to the body of knowledge into seasonal migration of elderly in two important ways. First, these findings provide a framework to estimate seasonal migratory patterns of the U.S. elderly population at the level of state-to-state transference. Second, these findings highlight the need for adjusting and fine-tuning public health and medical infrastructure necessary to provide critical care for those elderly patients. Public health and medical practitioners could use these findings to identify areas where and time when out-of-state elderly visitors may overwhelm the local infrastructures [19]. Suggested services to be evaluated include vaccination programs, hospital beds, home care services, and medical treatments for complications of P&I, particularly for resident and non-resident elderly, who face the most severe morbidity and highest mortality from these diseases.

Pneumonia and influenza prevention and treatment for elderly has never been more important. As of 2010, the U.S. has 46 million Medicare beneficiaries [20], most of whom are elderly. Exacerbating this situation are the rapid expansions of both size and proportion of the elderly population: As the large Baby Boomer cohort enters the age groups most vulnerable to the effects of influenza-associated morbidity and mortality, the impact of influenza will likely grow precipitously, resulting in an even greater, yet largely preventable strain on the already burdened health care delivery system [21]. A major strength of this analysis is the use of CMS data set, which is one of the most complete (96% coverage [22]) sources of information on U.S. elderly hospitalization profile. The recorded dates of admission permitted us to estimate the peak time of the outcome to the day level. Other in-depth analyses, such as relationship between hospitalizations with climatic features and holidays, can also be performed [23].

The analysis has some important limitations, however. First, the data used for the analysis are only a part of all insurance claims, and therefore do not represent the overall burden of P&I in the elderly population. Our cases likely represent only the more severe cases of influenza and its complications. Furthermore, Medicare covers approximately 96% of the elderly population. Therefore, the total number of P&I cases is likely an underestimate of the total P&I burden in the elderly [24].

Second, we defined the patients with out-of-state residential code as non-permanent residents, but we do not know whether non-residents of each state hospitalized for P&I in that state were living a substantial portion of the year in that particular year, or if they were in the state for a short period. There have been reports on elderly population keeping their original state identity while living in another state for a long term, mostly for tax-related benefits [25]. So, part of the increase in the ratios during winter could have been due to increase in seasonal migrants, while another part could have been due to those long-term stayers who have moved to a warmer place due to their deteriorating health while decided to keep their original state identity. Studies on whether snowbirds have less robust health compared to the national elderly are limited, and the results are inconclusive [26]. In either of the two circumstances, our findings still emphasize the importance to understand more on this non-residential hospitalized elderly population.

Despite the limitations outlined above, our study is among the first to quantify pneumonia and influenza hospitalization patterns in the elderly with respect to seasonal migration in the United States. Information on this seasonal migration patterns and influenza-associated disease patterns in this vulnerable population is critical in preparing for and controlling a potential influenza outbreak. We observed that nearly 15% of all P&I hospitalizations that occurred in Florida in peak influenza months were from patients visiting from another state. Influx of people can profoundly impact the health care system in destination states. The statistically significant delay of five days in the hospitalization peak time for the non-Floridian implies possible differences in susceptibilities or health seeking behaviors between the two groups or time and place of potential exposure to virus. The results of this and future related studies may explicate certain populations to target with public health interventions, such as vaccination, at the appropriate time to maximize effectiveness and reduce the burden of pneumonia and influenza in the elderly.

In future intervention studies, seasonally migrating elderly individuals could be universally vaccinated in their state of primary residence before the start of the influenza season to determine if this process could curtail the spread of influenza in their destination state. Additionally, flow mapping with multivariate visualization [27,28] and network analyses [29] show promise as valuable tools to quantify spatially and temporally how influenza dynamically flows among states to provide the most vulnerable populations the appropriate medical care and preventive measures.

## Conclusions

This study simultaneously assessed the spatial and temporal components of influenza-associated hospitalizations in the American elderly population, highlighting the seasonal patterns of influenza potentially related to seasonal migration of elderly individuals. Given the lack of national data on inter-state seasonal migration, we demonstrated the use of Medicare hospitalizations to quantify the impact of seasonal or temporary migration patterns on the distribution of pneumonia and influenza in the United States. Understanding where, when, and to whom hospitalizations occur is a critical component to predict, contain, or even prevent the spread of influenza to the vulnerable population of elderly adults, and will allow state and local health officials to plan for localized outbreaks and timely changes in health care services utilization.

## Competing interests

The authors have no specific financial interests, relationships, or affiliations relevant to the subject of this manuscript.

## Authors' contributions

KKHC led the writing process, executed the data visualization, and carried out the time-series analysis; SAC conceptualized the analysis, drafted the manuscript, and carried out the rest of the statistical analysis. Both KKHC and SAC contributed equally to the work. ENN purchased the Medicare data used in the analysis and provided substantive editorial feedback. All authors read and approved the final manuscript.

## Pre-publication history

The pre-publication history for this paper can be accessed here:

http://www.biomedcentral.com/1471-2458/11/444/prepub
